# Most nuclear systemic autoantigens are extremely disordered proteins: implications for the etiology of systemic autoimmunity

**DOI:** 10.1186/ar1832

**Published:** 2005-10-06

**Authors:** Philip L Carl, Brenda RS Temple, Philip L Cohen

**Affiliations:** 1 Department of Pharmacology, University of North Carolina, Chapel Hill, NC 27599, USA; 2 R. L. Juliano Structural Bioinformatics Core Facility, University of North Carolina, Chapel Hill, NC 27599, USA; 3 Division of Rheumatology, University of Pennsylvania School of Medicine and Philadelphia VA Medical Center, Philadelphia, PA 19104, USA

## Abstract

Patients with systemic autoimmune diseases usually produce high levels of antibodies to self-antigens (autoantigens). The repertoire of common autoantigens is remarkably limited, yet no readily understandable shared thread links these apparently diverse proteins. Using computer prediction algorithms, we have found that most nuclear systemic autoantigens are predicted to contain long regions of extreme structural disorder. Such disordered regions would generally make poor B cell epitopes and are predicted to be under-represented as potential T cell epitopes. Consideration of the potential role of protein disorder may give novel insights into the possible role of molecular mimicry in the pathogenesis of autoimmunity. The recognition of extreme autoantigen protein disorder has led us to an explicit model of epitope spreading that explains many of the paradoxical aspects of autoimmunity – in particular, the difficulty in identifying autoantigen-specific helper T cells that might collaborate with the B cells activated in systemic autoimmunity. The model also explains the experimentally observed breakdown of major histocompatibility complex (MHC) class specificity in peptides associated with the MHC II proteins of activated autoimmune B cells, and sheds light on the selection of particular T cell epitopes in autoimmunity. Finally, the model helps to rationalize the relative rarity of clinically significant autoimmunity despite the prevalence of low specificity/low avidity autoantibodies in normal individuals.

## Introduction

Why some proteins become autoantigens is one of the mysteries of immunology. Indeed, as Paul Plotz put it in a recent review, "The repertoire of target autoantigens is a *Wunderkammer *– a collection of curiosities – of molecules with no obvious linking principle" [1]. Most immunologists believe, probably with good reason, that making real progress in understanding and treating autoimmune diseases depends on solving this mystery.

While a single property might explain why these few proteins become autoantigens, it seems more likely that a combination of factors unites these proteins. Plotz divides such factors into four groups: structural properties, catabolism and fate after cell death, concentration and the microenvironment, and immunological and inflammatory properties. This paper will primarily deal with the first of Plotz's factors, the structural properties of autoantigens. Among the structural properties he lists are, citing the work of Dohlman and colleagues [2,3]: a highly charged surface, repetitive surface elements, bound nucleic acid, and the presence of a coiled coil. In this paper, we provide computational evidence that the first three of these properties can be understood as arising from the fact that most nuclear systemic autoantigens are extremely disordered proteins, and suggest that the fourth property, the presence of a coiled coil, occurs far less frequently than does disorder. We also show that several of the other factors mentioned by Plotz that may influence the selection of autoantigens also fit nicely into the picture of nuclear systemic autoantigens as extremely disordered proteins. We will argue that disordered proteins are apt to be poor activators of B cells for multiple reasons, and hence that B cells targeted to extremely disordered proteins are apt to escape immune deletion. Furthermore, because extremely disordered proteins tend to be highly sensitive to proteolysis and are predicted to have poor affinity for major histocompatibility complex (MHC) II, these proteins are also predicted to be under-represented as T cell epitopes. In the Discussion we propose a model of how the pool of potentially autoreactive B cells might subsequently become activated and lead to pathological consequences. This model explicitly incorporates the fact that, in addition to being disordered, the majority of nuclear systemic antigens are large complexes of highly expressed structural macromolecules. The model predicts that it should normally be difficult to identify T cell populations that activate autoimmune B cells, and that such activation might not require cell-to-cell contact between B and T cells. Considerable evidence supports both of these predictions. At the same time the model explains why, paradoxically, some type of T cell-B cell contact is required in the development of autoimmunity. Finally, the model provides insights into why a specific T cell epitope is most commonly associated with the SmB autoantigen in systemic lupus erythematosus (SLE).

### Defining protein disorder

The dominant picture of protein structure is that proteins fold to a unique native state of lowest energy. There is now an increased appreciation that the native state may not be a single structure after all, but rather an ensemble of closely related structures [4,5]. More recently has come an appreciation that large regions of some proteins never fold at all, at least in the absence of a binding partner. Regions that lack a fixed tertiary structure as determined by weak or missing electron density in a solved X-ray structure are identified as intrinsically disordered. In what follows we shall use the terms 'disordered protein' and 'disordered region' somewhat interchangeably, while recognizing that a 'disordered protein' can have regions of extensive order. It is important to distinguish between a disordered region that has a multiplicity of structures and a region such as a loop that lacks alpha-helical or beta-sheet secondary structure but may exist in a single structure.

While some aspects of protein disorder were appreciated more than 50 years ago, we can thank Dunker and Obradovic and their colleagues [6] for the current renaissance of interest in the concept. A more rigorous discussion of the concept of protein disorder is provided by Dunker *et al*. [6,7]. Excellent recent reviews of protein disorder are provided by Uversky, Gillespie and Fink [8], Fink [9], and Dyson and Wright [10], who call such proteins 'natively unfolded' or 'intrinsically unstructured'.

To develop software capable of predicting disordered regions, Dunker, Obradovic and their colleagues analyzed experimentally determined structures with disordered regions. They developed a neural network model to predict disorder, trained on regions of missing electron density in X-ray structures and disordered regions in NMR structures. The current default PONDR^® ^predictor at the PONDR^® ^web site [11] is VL-XT [12-14]. It is a hybrid of three earlier predictors: VL1 used for internal regions starting and ending 11 residues from the protein terminus; XN, an amino terminus predictor; and XC, a carboxyl terminus predictor. These predictors use a variety of input attributes including coordination number, net charge, hydropathy, and the presence of particular combinations of amino acids. The false positive error rate, that is, the prediction of disorder when a region is known to be ordered, of the VL-XT predictor is estimated at 22% on a per residue basis. However, the predictor is far better at predicting long regions of disorder, so that the false positive rate per residue drops to 1.7% per residue for consecutive regions of predicted disorder ≥40 residues. Further details on the training and accuracy of the various PONDR^® ^predictors are available on the PONDR^® ^web site.

Some additional PONDR^® ^predictors are available at DisProt [15], but these have not been used in this study.

PONDR^® ^scores are characterized by a disorder index q, which can range from 0 to 1, and are averaged over a window of nine amino acids. The boundary between order and disorder is conventionally set at q = 0.5. There is no clear criterion for extreme disorder. In this paper we call a protein extremely disordered if it contains at least one long disordered region (LDR) of 39 or more consecutive residues as predicted by PONDR^®^.

One should note that there are now several other web-based predictors of protein disorder available based on different algorithms and training sets. Examples are the DISOPRED [16] and DISEMBL™ [17] predictors. DISEMBL™ also has a complementary program GlobPlot™ [18] that focuses on predicting order. For the 19 LDRs presented in the figures, we have also determined the degree of disorder using the two DISEMBL™ and the DISOPRED disorder predictors. For all the predictors, on average 57% to 70% of the residues in the LDR predicted by PONDR^® ^were confirmed to be disordered. This agreement suggests that our conclusions about LDRs are not strongly dependent on the particular disorder predictor used.

## Materials and methods

A database of 51 nuclear systemic autoantigens (hNuSysAAG) was generated by SWISS-PROT text searches using SRS [19] combined with literature searches for autoantigens not yet annotated in SWISS-PROT. Keywords used in searching SWISS-PROT included 'human (organism) and nuclear and (autoantigen or autoimmune or antigen)' or 'human (organism) and nuclear and (scleroderma or sclerosis or lupus or sjogren)'. In a few cases, for example, the histones, we added widely recognized systemic nuclear autoantigens that were not annotated as autoantigens in SWISS-PROT. Proteins were removed from the initial search results for the following reasons: non-nuclear subcellular location (although it is not always clear how to classify the cellular location of a protein that is largely located in the cytoplasm, such as Ro 52K, but that shuttles to the nucleus – we generally assigned a nuclear location to such proteins despite the degree of ambiguity involved); not related to a systemic autoimmune disease; origin in a complex that was autoantigenic, but the protein was not autoantigenic itself. Three additional control databases were generated from SWISS-PROT: 10,962 human proteins (hSP); 2,335 human nuclear proteins (hNuSP); and 8,627 human non-nuclear proteins (hNNuSP).

All the predictions of order/disorder presented in this paper were made with the VL-XT predictor available at the PONDR^® ^web site [11]. The predictions of class II dependent T cell epitopes were made with the ProPred predictor [20].

## Results

### Most nuclear systemic autoantigens are predicted to contain extremely disordered regions

**Figure 1:**
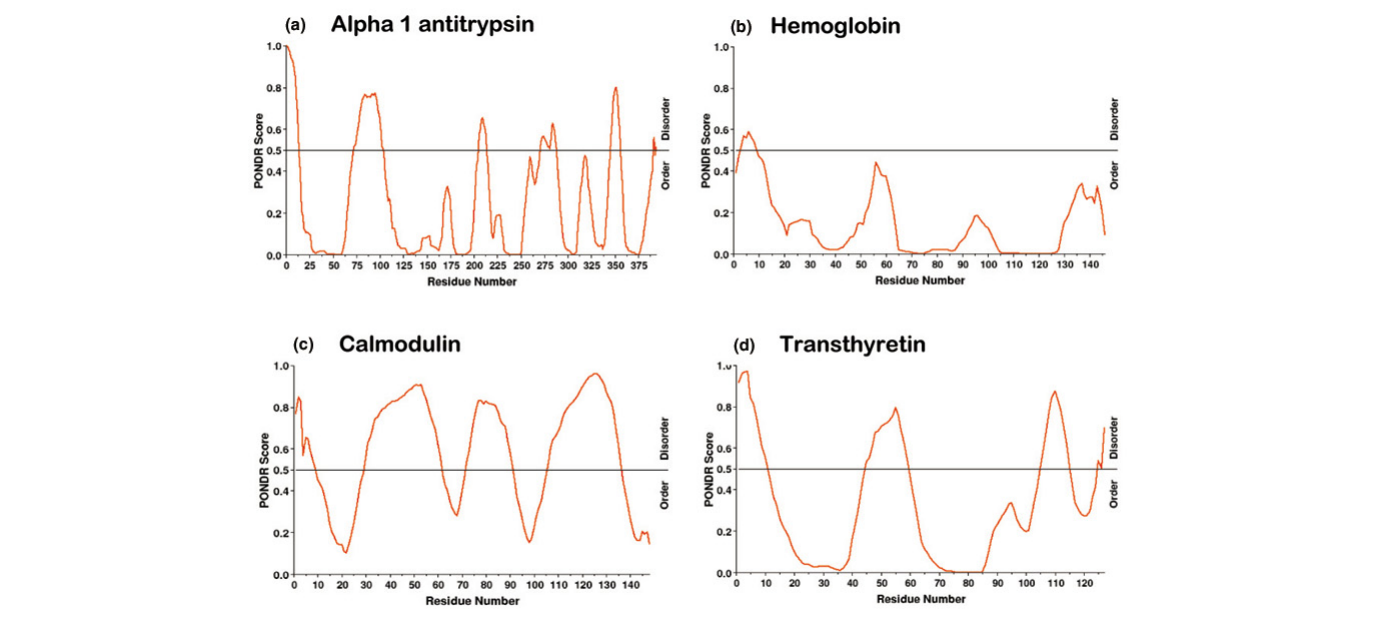
PONDR^® ^predictions of disorder for four familiar human proteins. The SwissProt Accession Numbers [63] are given in parentheses. **(a) **Alpha-1-antitrypsin (P01009); **(b) **hemoglobin B (P02023); **(c) **calmodulin (P62158); **(d) **transthyretin precursor human (P02766). The line at PONDR^® ^score 0.5 defines the disorder threshold and is an arbitrary measure used to distinguish order from disorder. The PONDR^® ^predictor used here and in all other diagrams in this paper is VL-XT, which is the default predictor on the PONDR^® ^web site.

**Figure 2:**
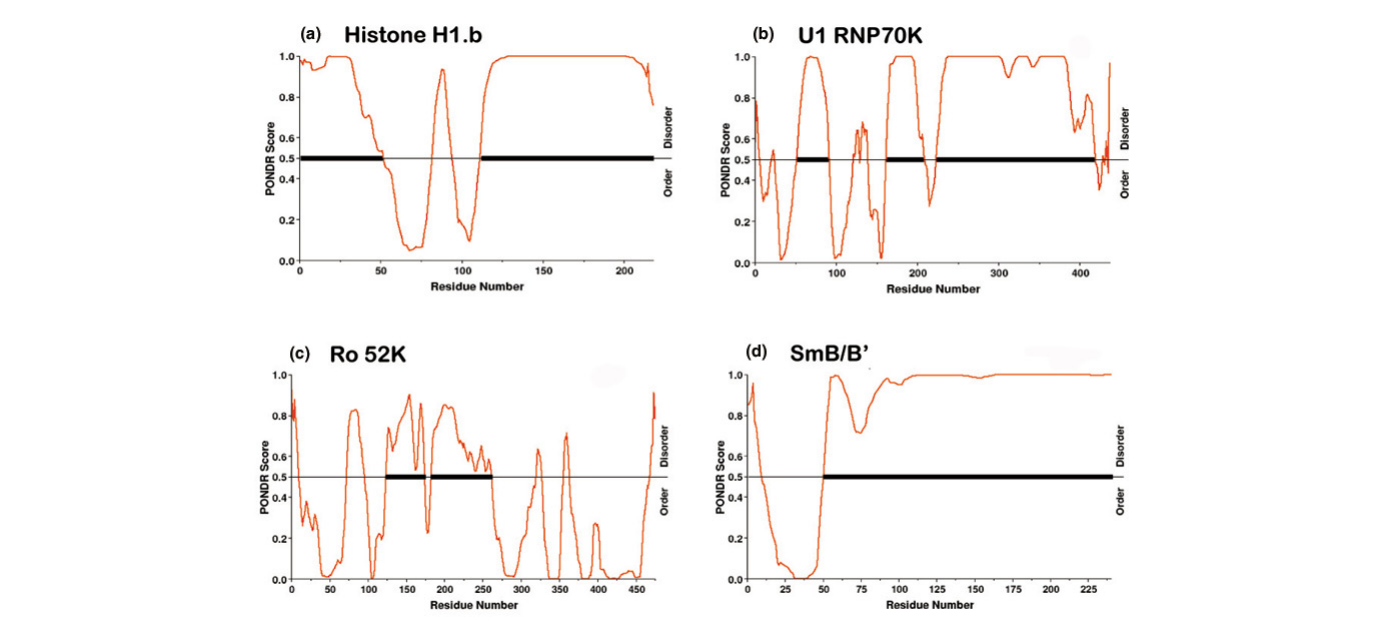
The PONDR^® ^plot of several autoantigens selected from Table 1 (Additional file 1). The proteins shown are: **(a) **histone H1b (P10412); **(b) **U1 RNP70K (P08621); **(c) **Ro 52K (P19474); **(d) **SmB/B (P14678). The heavy horizontal black bars indicate regions of 39 or more successive disordered residues with a PONDR^® ^score greater than the threshold of 0.5.

PONDR^® ^predictions for proteins vary from highly ordered to almost completely disordered. In Fig. 1 we show typical patterns for several human proteins, none of which are known autoantigens, and all of which are in the Protein Data Bank (PDB) [21], a structural database that is known to contain largely ordered proteins. In contrast, the PONDR^® ^plots of several nuclear systemic autoantigens are shown in Fig. 2. It is clear that the autoantigens shown in Fig. 2 are predicted to be far more disordered than the non-autoantigenic proteins shown in Fig. 1. To gain insight into the significance of the relationship between disorder and autoantigenicity, we performed analyses of the various databases described earlier.

Of the 51 autoantigens in our hNuSysAAG database, 76% of the proteins met our criterion for extreme disorder, which was comparable with 75% of the proteins in hNuSP. In contrast, only 49% of hSP and 42% of hNNuSP met our criterion for extreme disorder. Thus, while nuclear autoantigens are no more disordered than nuclear proteins as a whole, nuclear proteins in general are significantly more likely to be disordered than non-nuclear proteins. It is interesting to note that 50% of the proteins annotated in SWISS-PROT as autoantigens are nuclear proteins but only 21% of human proteins are nuclear, implying disorder may play a role in this enrichment of nuclear proteins as autoantigens.

Our results can be compared to a recent paper by Iakoucheva *et al*. [22] that demonstrated that proteins associated with cancer (79% of proteins) and proteins associated with signal transduction (66% of proteins) are more highly disordered than the typical eukaryotic protein in the SWISS-PROT database (47% of proteins) or the PDB (13% of proteins). Note that these authors have defined a long disordered region as 30 or more residues compared with our criterion of 39 or more residues. Using Iakoucheva *et al*.'s criterion, we found that 83% of the proteins in hNuSysAAG met the requirement for the long disordered region. Thus, the proteins in hNuSysAAG are at least as disordered as the cancer-associated and signaling proteins studied by Iakoucheva *et al. *[22].

Some additional evidence also suggests that disorder and autoantigenicity are linked. In particular, the most common autoantigens in the Sm particle are Sm B/B', Sm D1 and Sm D3. All three proteins contain a long disordered region ≥39 consecutive residues. In contrast, a PONDR^® ^analysis of Sm E, Sm F, and Sm G, proteins in the Sm particle that are rarely if ever autoantigens, lack long disordered regions (data not shown).

### Experimental evidence that nuclear systemic autoantigens are extremely disordered proteins

Certain experimental evidence suggests that most nuclear systemic autoantigens are indeed, as predicted, disordered. For example, the La autoantigen is known to be especially sensitive to proteolysis consistent with a disordered structure [23,24]. The amino terminus of DNA topoisomerase I has been shown to be disordered by limited proteolysis [25], circular dichroism and gel filtration [26]. Furthermore, the positively charged tails of the histones are proteolytically sensitive and are not observed to contribute electron density [27].

**Figure 3:**
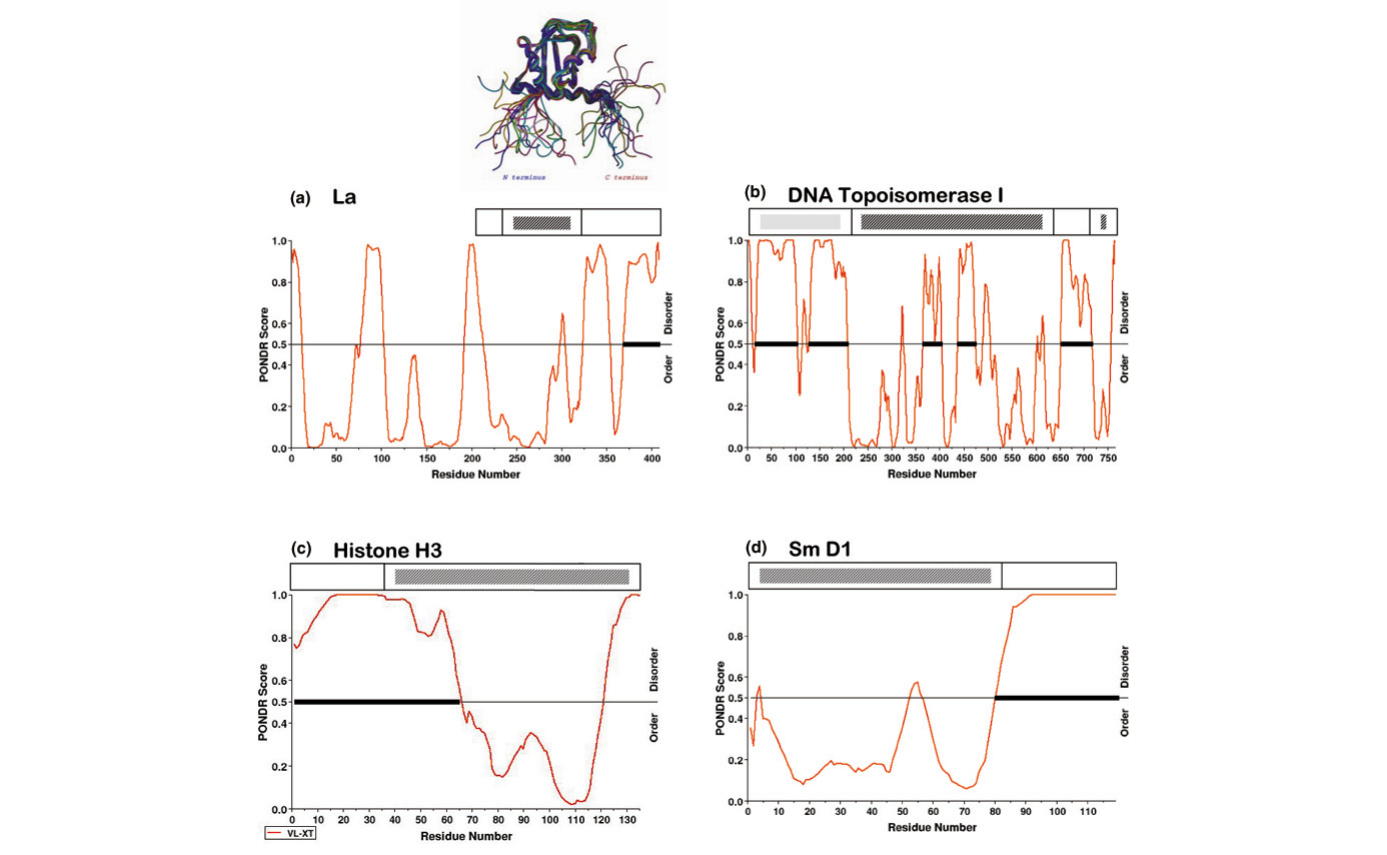
PONDR^® ^predictions compared to experimental structural determinations for various autoantigens. **(a) **La autoantigen (Swiss-Prot: P05455). The shaded box above the plot (residues 231–325) is the region that Jacks *et al*. [64] determined to be ordered via NMR. The empty boxes (residues 214–230 and residues 326–408) are regions determined to be unstructured or disordered. The inset (PDB: 1OWX; La222-334) shows the conformational flexibility of disordered regions at the amino and carboxyl terminii of the La fragment. **(b) **DNA topoisomerase I (Swiss-Prot: P11387). The structure was determined by X-ray methods for a protein-DNA complex (PDB: 1EJ9) encompassing residues 203–765 of DNA topoisomerase I. Residues 634–713 (empty box) are missing and, therefore, disordered in the structure [65]. The lightly shaded box at the amino terminus is the region that was determined to be disordered in the references cited above. **(c) **Histone H3 (Swiss-Prot: P68431). The structure of chicken H3 in a histone octamer complex (PDB: 2HIO) was determined by X-ray methods for residues 1–135. Residues 1–42 are missing, presumably due to disorder [66]. **(d) **Sm D1 (Swiss-Prot: P62314). The structure of a protein complex between Sm D1 (residues 2–119) and Sm D2 was studied by X-ray methods (PDB: 1B34) [67]. Residues 82–119 from Sm D1 are missing from the structure.

In general, it is difficult to crystallize extremely disordered proteins. Thus X-ray studies of extremely disordered proteins tend either to focus on the ordered domains of the proteins that can be readily crystallized, or are studies of protein complexes where some disordered domains become ordered on binding. While NMR studies are not restricted to proteins that can crystallize, only small proteins are readily amenable to NMR methods so that often only domains of larger proteins are studied. Despite these limitations, direct evidence illustrated in Fig. 3 indicates that PONDR^® ^predictions of disordered regions correlate well with structural determinations for several nuclear systemic autoantigens.

The fact that the structural studies in each of these cases stop close to the predicted boundary between order and disorder strongly suggests that the indicated regions have been correctly identified as disordered by PONDR^®^. Some of the disparity between prediction and experiment may be explained by complex formation. For example, in topoisomerase I, PONDR^® ^predicts disorder from 365–404 and 437–475 whereas structures of topoisomerase I in complex with DNA show these regions are ordered. These residues possibly act as linkers connecting domains of topoisomerase I that interact with opposite sides of the DNA; they may be unstructured in the apoprotein and become ordered upon binding DNA.

### Properties of disordered proteins of relevance to the nature of autoantigens

The amino acid composition of disordered regions is distinct from that of ordered regions [6]. Typically disordered regions are deficient in Trp, Cys, Phe, Ile, Tyr, Val, Leu, and Asn. They are enriched in Ala, Arg, Gly, Gln, Ser, Pro, Glu, and Lys. This bias in amino acid composition is reflected in the fact that disordered regions typically have a strong net charge, which is the first attribute of autoantigens mentioned by Plotz [1]. One consequence of this skewed amino acid composition of disordered regions is that many strongly disordered regions have very low sequence complexity as measured by Shannon's entropy [13], which can in turn lead to a preference for repetitive surface elements, the second of Plotz's factors thought to influence autoantigen structure. (However, not all regions of low sequence complexity are disordered.) The low sequence complexity of autoantigens is readily observed using a Web-based tool such as the GlobPlot™ server [18]. Although statistics on the fraction of all proteins that contain segments of low complexity are not readily available, we note that of 24 low complexity regions found in 13 of the most common nuclear systemic autoantigens, all but two occur in regions of disorder as determined by PONDR^® ^(data not shown).

Many functions have been ascribed to disordered proteins [7], but one of the most prominent is binding to nucleic acid [7,10]. This is also a factor mentioned by Plotz as a third characteristic of the structure of autoantigens. In addition, recent work [28] shows that sites of phosphorylation are correlated with sites of protein disorder. Because phosphorylation/dephosphorylation are factors mentioned by Plotz as likely to be important in the selection of autoantigens [1], this is one more piece of evidence, albeit indirect, that disorder is apt to play a role in this process. The fourth structural criterion characteristic of autoantigens noted by Plotz (citing Dohlman *et al*. [2]), is the predicted presence of a coiled coil. The mechanism by which coiled coils may promote antigenicity is unclear, but Howard *et al*. [29] showed that a region at the amino terminus of the autoantigen histidyl-tRNA synthetase (which Coils [30] predicts to be a strong coiled coil (data not shown)) may promote autoimmunity by activation of dendritic cells. When we examined our database of nuclear systemic autoantigens using the Coils predictor, we found that coiled coils were present in 29% of our proteins whereas long disordered regions were present in 76% of our proteins. (Dohlman *et al*. [2] report a value of 36.7% coiled coils in their database of systemic autoantigens compared to 8.7% in the SwissProt and 1.1% in the PDB.) Thus, in agreement with Dohlman *et al*. [2] coiled coils appear to be over-represented in our collection of nuclear systemic autoantigens. Coiled coils are predicted roughly as frequently in our autoantigens that have long disordered regions as in the minority that do not. However, it is interesting to note that the most frequently encountered nuclear systemic autoantigens, such as the histones, the Sm proteins, and the U1 and centromere binding proteins, are all completely devoid of predicted coiled coils and are extremely disordered. (It should be noted that Dohlman *et al*. [2] stated that U1 snRNP70K and CENB possessed coiled coils. However, using an updated version of the Coils predictor that was unavailable to Dohlman *et al*., we found that these two predictions were in error. When the predictions were run using additional weighting of the amino acids appearing in positions 1 and 4 of the heptad repeat, which helps to rule out false positives, we were unable to confirm the putative coiled coils.)

In some cases, a region predicted by PONDR^® ^to be disordered overlaps with a region predicted by Coils to be a coiled coil. An example is Ro 52K. Here the two disordered regions are predicted to be 124–174 and 183–261; the predicted coiled coils cover 128–165 and 189–234. Ottosson *et al*. [31] present experimental evidence showing the peptide 200–239 'had a partly α-helical secondary structure with major contribution of random coil,' that is, both the Coils and the PONDR^® ^predictor seemed to be partially correct. In summary, we have confirmed the results of Dohlman *et al*. [2] that coiled coils seem to be common in autoantigens, but there is currently no evidence that this conclusion conflicts with the prediction that nuclear systemic autoantigens are disordered.

### Disordered regions are predicted to make poor T cell antigens

B cells generally require T cell help to become activated and secrete their antibody product. Although T cells are required for the production of antinuclear autoantibodies in multiple animal models and probably also in humans, it has been notoriously difficult to isolate nuclear antigen-reactive T cells and to explore their specificity and function. We examined the predicted ability of several nuclear systemic autoantigens to function as T cell epitopes (when presented by MHC class II molecules) and asked if these sequences resided in areas of disorder; we used the web server ProPred [20,32]. This site implements the computer program TEPITOPE, which predicts peptide sequences that offer promise as promiscuous T cell epitopes [33]. The available evidence, though limited, suggests that TEPITOPE predicts many sequences that are experimentally verified T cell epitopes, although it also predicts many sequences to be T cell epitopes that cannot be verified as such [34-36]. This latter point is hardly surprising as TEPITOPE's predictions are based solely on binding to MHC II and do not attempt to model cellular compartmentalization of the antigen and specific proteolysis of the protein. The most extensive analysis [37] suggests that at least 50% of TEPITOPES predictions are verifiable, although the data also suggest that predictions for certain MHC alleles may be more accurate than others. We wondered if disordered regions might be particularly poor candidates for strong binding to MHC II proteins and, therefore, unlikely to be T cell epitopes.

**Figure 4:**
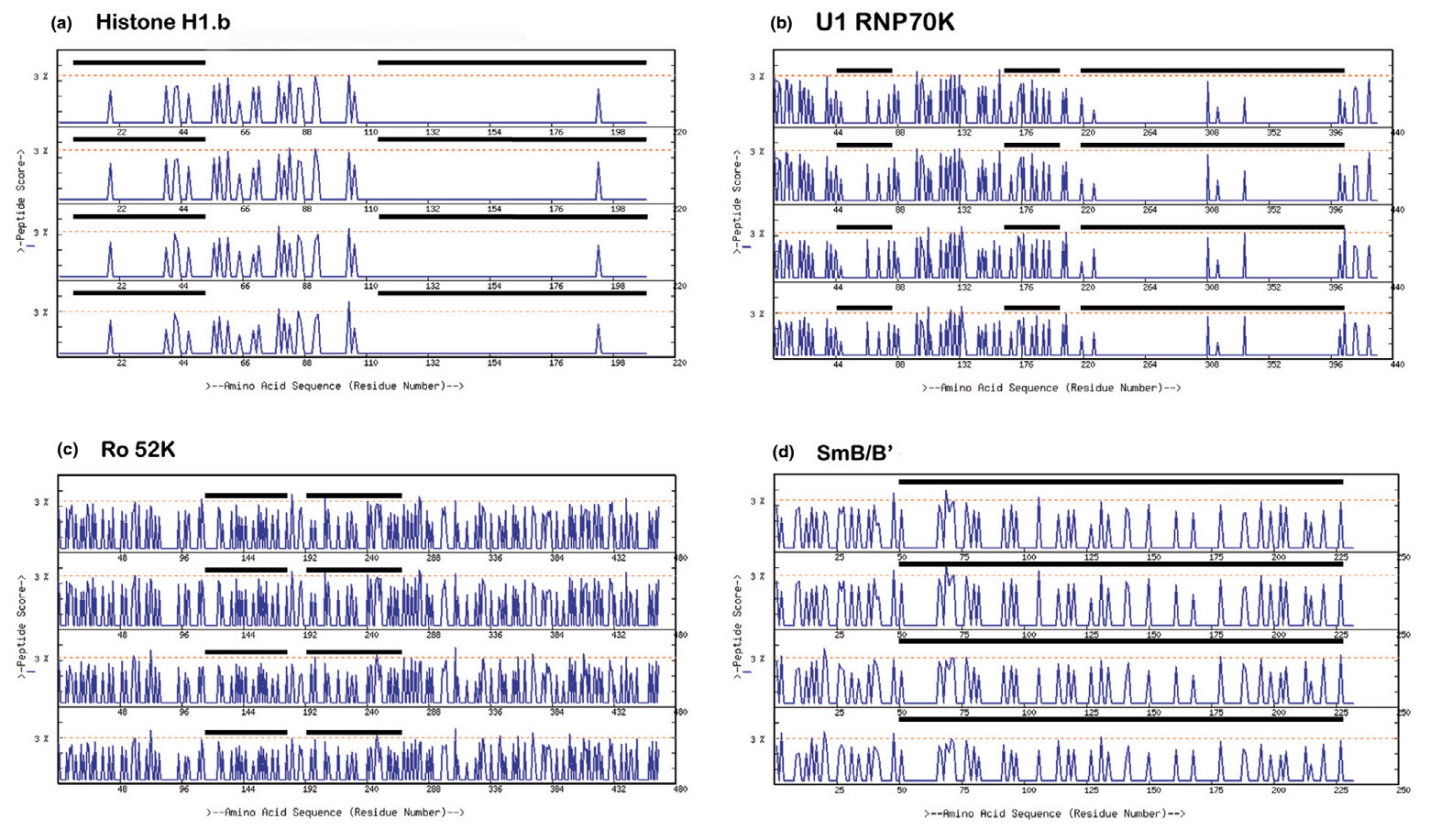
T cell epitopes for several autoantigens predicted by the ProPred server. **(a) **histone H1b (Swiss-Prot: P10412). **(b) **U1 RNP70K (Swiss-Prot: P08621). **(c) **Ro 52K (Swiss-Prot: P19474). **(d) **Sm B/B' (Swiss-Prot: P14678). Only four alleles are shown for each protein for the HLA antigens (from the top down): DRB1_0101; DRB1_0102; DRB_0301; and DRB1_0305. The patterns for the remaining MHC II alleles follow the same general trends. The black bars highlight the long disordered regions of the sequence as pictured in Fig. 2. The horizontal dotted red line is the threshold score-here set at the default value of 3%, which is used to differentiate between binders and non-binders. A threshold of 3% means that the protein sequence belongs to the 3% best scoring natural peptides. The lower the threshold percentage the fewer false positive peptides will be predicted to be T cell epitopes.

**Figure 5:**
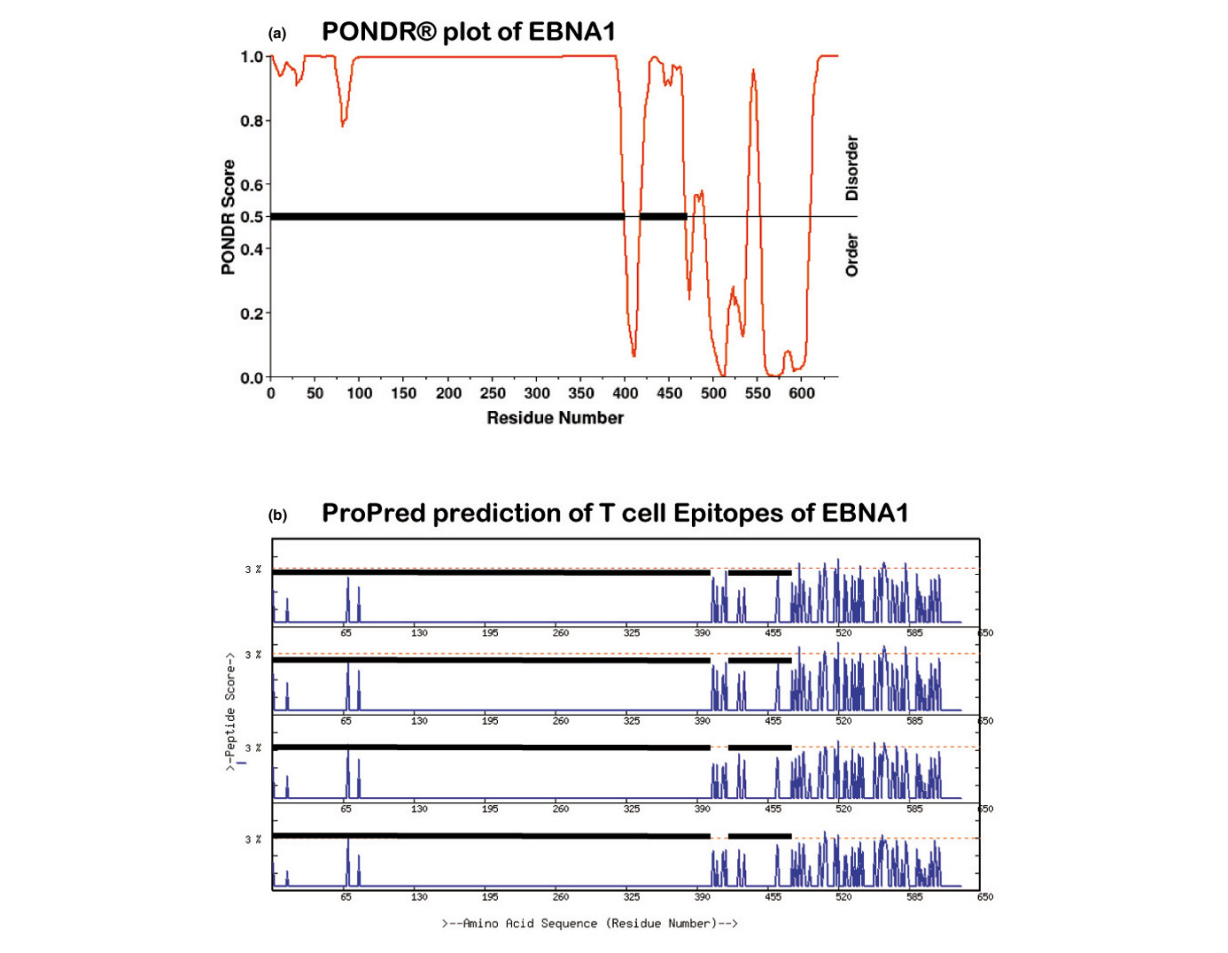
Disorder and T cell epitope prediction for EBV Nuclear Antigen 1. **(a) **PONDR^® ^plot of the Epstein Barr Nuclear Antigen 1 protein (Swiss-Prot: P03211). The PPPGRPP epitope that induces cross-reactivity to an epitope on Sm B/B' is found in residues 398–412, almost exactly at the sharp minimum of the PONDR^® ^plot. This is the only known cross-reacting epitope in the virus. **(b) **T cell epitopes of EBNA1 predicted by the ProPred server. Only the results for alleles HLA-DRB_01, HLA-DRB_0102, HLA-DRB1_0301, and HLA-DRB_0305 are shown. The remaining 47 alleles show a very similar picture. The threshold is set at 3%. The black bars delimit the strongly disordered regions of the PONDR^® ^plot shown in (a). It is apparent that the highly disordered region of the first approximately 400 amino acids is predicted to be nearly devoid of potential T cell epitopes. The epitope from residues 398–412 that cross-reacts with the SmB protein is predicted to be most reactive with alleles HLA DRB5_0101 and DRB5_0105, although just slightly below a 3% threshold (data not shown).

Representative results for several HLA-DR alleles are shown in Fig. 4. If one compares the overall pattern of PONDR® predictions from Fig. 2 with the T cell antigen prediction from Fig. 4, one can see that the strongly disordered regions of the PONDR^® ^plots correspond to regions of the T cell epitope plot in which only a very few even potential epitopes are located. By a potential epitope we mean epitope represented by a peak in the ProPred output without necessarily considering whether that peak is above the threshold. In fact, the vast majority of the potential epitopes illustrated in Fig. 4 are below threshold and, therefore, would not be predicted to be epitopes. For reasons of space we only show the results for four alleles and the four autoantigens whose PONDR^® ^plot was displayed in Fig. 2. For example, for Histone H1b in Fig. 2a the PONDR^® ^plot shows strong disorder in the region from residues 1–51 and from 112–218. The former region in Fig. 4a is somewhat depleted of potential T cell epitopes and the latter nearly devoid of potential epitopes. For U1 RNP70K the PONDR^® ^plot in Fig. 2b shows strong regions of disorder at residues 52–91, 162–209, and 224–418. Although there still appear to be some possible epitope candidates in the former two regions in Fig. 4b, the latter region is again nearly devoid of potential epitopes. In the PONDR^® ^plot of Fig. 2c, the disordered regions of Ro 52K from 124–174 and 183–261 can readily be seen to correspond to a slight diminution in the frequency of prospective epitopes in Fig. 4c. While the effect here is far less dramatic than in the case of the three other autoantigens pictured, the degree of disorder seen in Fig. 2 for Ro 52K is considerably less than for the other autoepitopes. Finally, the strongly disordered region in Sm B/B' from residues 51–240 in Fig. 2d corresponds to a marked deficit of potential T cell candidates in the same region in Fig. 4d compared to the number of potential epitopes in the first 50 residues. An even more dramatic demonstration of the correspondence of regions of extreme disorder and a lack of potential T cell epitopes will be discussed in Fig. 5. Taken together, these data suggest that disordered regions, probably because of their conformational flexibility, masking by nucleic acids and other proteins and their proteolytic lability, make poor antigens. Thus, both intuitions about what makes a good antigen and the computational analysis of predicted MHC II T cell epitopes support the notion that there will be few T cells targeted to extremely disordered regions. Proteins with extensive regions of disorder are thus likely to elicit poor T cell responses. B cells reactive against these nuclear antigens are unlikely to receive cognate help, and would be neither activated nor deleted. These clones thus represent a potential source of autoreactive antibodies.

### Autoantibodies recognize both ordered and disordered regions

Given that clones targeted to extremely disordered proteins are a potential source of autoimmune antibodies, it is natural to wonder if in fact one can subsequently detect autoantibodies directed against the disordered regions. The obvious way to explore this question is to compare epitope maps for some common autoantigens with the maps of disordered regions provided by PONDR^®^. This exercise is, however, more difficult than it might seem. For example, Moutsopoulos *et al*. [38] have reviewed the epitope mapping data for Ro 60 kD, Ro 52 kD, and La 48 kD. It is apparent from their paper that different groups using different techniques on different patient samples have identified different linear epitopes and that, for many of the autoantigens, most of the protein sequence has been identified as an autoepitope by one group or another. Nonetheless, one can ask if disordered regions ever appear as autoepitopes. The answer is a clear yes. For example, in Ro 52K multiple authors have located an autoepitope at residues 216–292. Much of this epitope overlaps with the predicted strongly disordered region in Ro 52K from residues 183–261 (see Fig. 2c). Similarly, autoantigen La shows a predicted strongly disordered region from residues 369–408, which is another region targeted by autoantibodies. Many other B cell epitopes to Sm B have been located largely at the carboxyl terminus of the protein [39]. As is readily seen in Fig. 2d, this region of the protein is predicted to be largely disordered. Furthermore, linear epitope mapping may not be finding the most relevant conformational epitopes. So while it is clear that many epitopes on autoantigens are located in disordered regions of the antigen, it is also true that large regions of autoantigens are often autoepitopes, rendering any correspondence between disordered regions and autoepitopes less than convincing.

### Protein disorder and epitope spreading

Spreading describes the extension of immune reactivity from an initial region of strong antigenicity towards a polypeptide into other epitopes of the autoantigen, or even from an epitope in one polypeptide to another polypeptide in a macromolecular complex such as the nucleosome or the Sm particle [40,41]. Spreading can lead to a more rapid and intense secondary response, longer lasting immune memory and multiple other advantages [40]. In a disease such as SLE, the reactivity can even extend into a different type of macromolecule such as DNA or RNA. Judith James and her colleagues have carried out several elegant experimental demonstrations of spreading. In a key study [42] they showed that immunization of rabbits with the peptide PPPGMRPP, a repeated sequence within the carboxyl terminus of Sm B/B', led to a spreading of the B cell response to many different structures on the SmB/B' autoantigen. A salient observation was that the antibodies reactive against these secondary determinants were in general not cross-reactive with the initiating peptide. In subsequent work [43], these authors showed that the closely related peptide PPPGRPP found in the nuclear antigen 1 (EBNA1) of the Epstein-Barr virus (EBV) was also capable of eliciting a lupus-like disease in rabbits. This result is of great interest given the evidence that the authors cite that EBV may be an etiological agent of autoimmune disease. A reasonable hypothesis is thus that EBV might attempt to circumvent immune surveillance by utilizing molecular mimicry. The subsequent attempt to deal with an EBV infection might lead to an autoimmune attack, initially on similar sequences in the B/B' polypeptide followed by spreading to the rest of the Sm particle.

To further explore the relevance of disorder to the idea of spreading we carried out a PONDR^® ^analysis of the EBNA1 protein. The results are shown in Fig. 5. The results shown in Fig. 5a extend the notion of molecular mimicry [44] by suggesting that the EBNA1 protein has evolved to present, as nearly as possible, a disordered face to the immune system. The PPPGRPP epitope is one of the few regions of the protein that is relatively ordered, and because it mimics a self-antigen of Sm B/B' the immune system has a difficult job in defending against EBV infection. An antibody response against the ordered epitope risks subsequent development of autoimmune disease because the same spreading, which presumably allows defense against the disordered regions of EBNA1, carries the risk of a similar spreading to other epitopes in the Sm particle.

This view of the battle between the virus and the immune system is further amplified by the results of the analysis of MHC II T cell epitopes using the ProPred server shown in Fig. 5b. Here we can see that the extremely disordered regions of the virus contain essentially no predicted T cell epitopes in the context of MHC II. This is further strong evidence that a suspected pathogen implicated in autoimmune disease has escaped immune surveillance by using disorder to 'fly below' the level of sensitivity of the T cell receptor. Thus the virus seems to use both disorder and molecular mimicry as part of the infectious process. There have been earlier suggestions that protein disorder may allow viruses or presumably other pathogens to evade immune detection [45,46]. While the above example supports the notion of molecular mimicry as an important process in the development of autoimmune disease, we do not wish to suggest that other mechanisms that might lead to autoimmunity have been ruled out. Indeed, it seems that defects in apoptosis allowing exposure of cryptic disordered antigens to the immune system might be an important mechanism in many cases [12,47,48].

**Figure 6:**
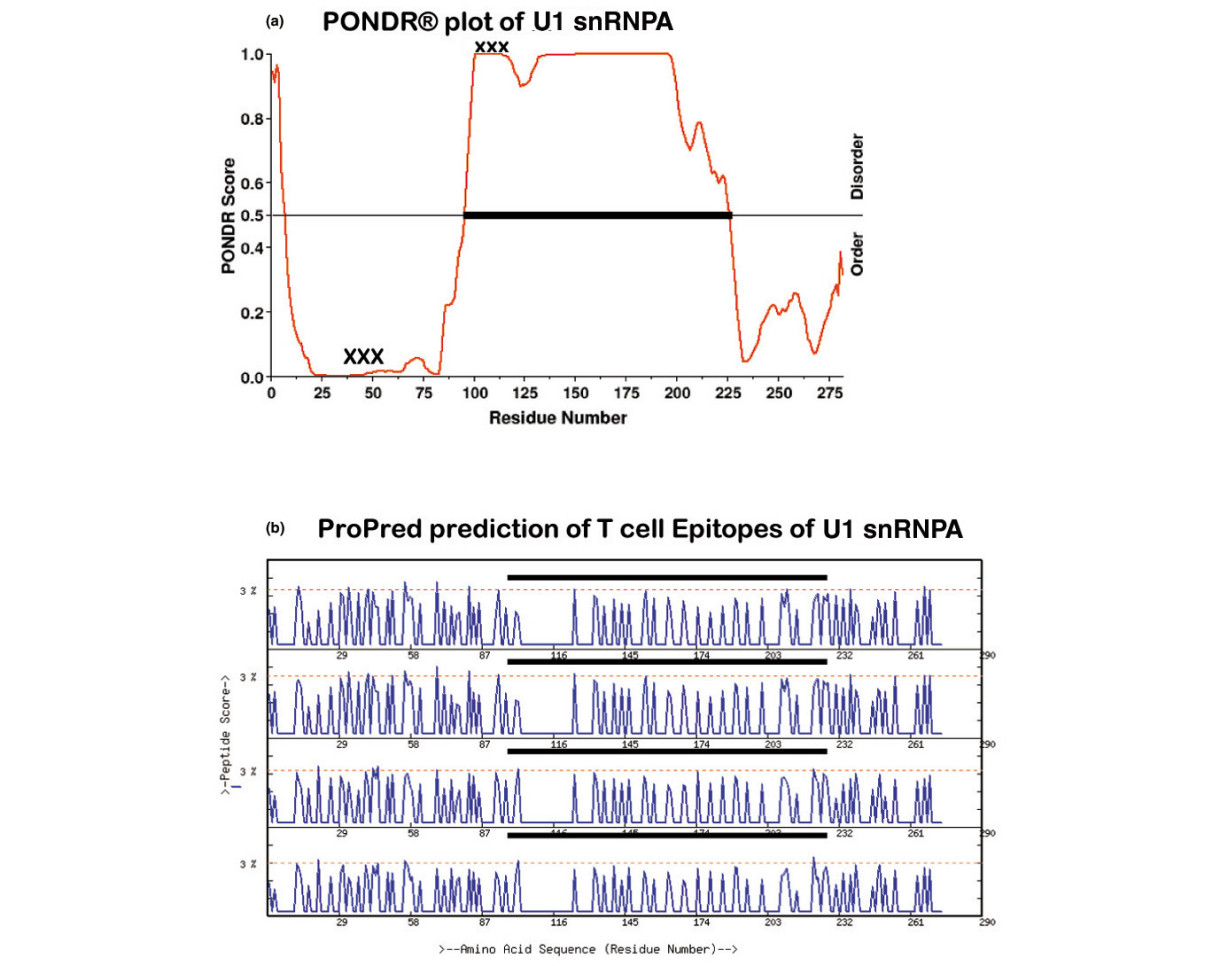
Disorder and T cell epitope for U1 snRNPA. **(a) **PONDR^® ^plot of the U1 snRNPA protein (Swiss-Prot: P09012). The location of the strongly immunogenic peptide A3 (residues 44–56), which induces spreading and systemic autoimmune disease, is indicated by XXX. The weakly immunogenic peptide A6 (residues 103–115), which does not induce spreading or autoimmune disease [49], is indicated by xxx. **(b) **ProPred analysis of the U1 snRNPA protein in the context of MHC II. Only the results for alleles HLA-DRB_01, HLA-DRB_0102, HLA-DRB1_0301, and HLA-DRB_0305 are shown. The remaining 47 alleles show a very similar picture. The threshold is set at 3%. The black bar delimits the long disordered region of (a).

As another example of how a consideration of protein disorder can shed light on the phenomenon of spreading we consider further work from James' group [49]. They examined the immunogenicity and antigenicity in rabbits of two strong epitopes of the lupus autoantigen small nuclear ribonucleoprotein particle U1 snRNPA protein (also known as the U1A protein). One peptide, A3, was a strong immunogen, and in the months following initial immunization antibodies against this peptide exhibited spreading to other common epitopes of U1 snRNPA. In contrast, the A6 peptide was a weaker immunogen, and antibodies to this epitope do not show spreading. Not only was spreading associated solely with the A3 epitope, but also this epitope, unlike the A6 epitope, was able to induce clinical signs of autoimmune disease such as leukopenia and renal insufficiency. The authors asked why these two epitopes, located fairly close together in the same polypeptide, exhibit such different immunological and pathological properties. They point out that the two peptides have similar high isoelectric points, which are fair indicators of antigenicity in the snRNP system, and that A6, like some other autoimmune epitopes, is relatively non-immunogenic. It may be significant that, as shown in the PONDR^® ^plot in Fig. 6, the A3 epitope that is capable of inducing spreading and autoimmune disease like the EBNA1 epitope shown in Fig. 5, is in a strongly ordered region located adjacent to regions of strong disorder of the PONDR^® ^plot. In contrast, the A6 epitope is in a region of strong disorder. Once again in support of these notions, we have carried out an analysis of the predicted T cell epitopes in these regions. The results shown in Fig. 6b confirm a paucity of T cell MHC II epitopes in the extremely disordered region 96–226. In particular, there are few even potential T cell epitopes predicted in the region from 103–115 where the A6 peptide is located.

Recent work on the mechanism of spreading from Gordon, McCluskey and colleagues [50] extending their earlier studies of the Ro/La system [51,52] suggest that one can obtain an antibody response to several regions of the La autoantigen following immunization with recombinant La. In contrast, when they immunized with Ro 52K or Ro 60K, the only region of La in which spreading was seen to occur was the carboxy-terminal region which, as shown in Fig. 3a, is the only region of La that is strongly disordered. These results are again consistent with the pattern of spreading moving from ordered to disordered regions.

## Discussion

Any theory of autoimmunity needs to account for at least two observations. The first is of the existence of large numbers of self-reactive immune cells, normally deleted or inactivated during tolerization, with specificity for a limited number of autoantigens. The second is that having escaped destruction, these immune cells can somehow subsequently become activated. The appreciation that many nuclear autoantigens are disordered can shed light on possible mechanisms by which both of these events can occur.

*A priori *one might expect the disordered regions of proteins to be poor antigens. By definition they exist in multiple conformations, which would suggest that it would be difficult to develop a conformation-specific antibody against such a region. In addition, disordered regions are very sensitive to proteolysis [7]. Furthermore, because disordered regions are often bound to other proteins or to nucleic acids, they may be masked and physically unavailable to the immune system [49]. Finally, as shown by the ProPred analysis, disordered regions are only rarely apt to be T cell epitopes. In summary, the recognition that most nuclear systemic autoantigens contain long disordered regions goes a long way towards explaining why a pool of potentially autoreactive B cells, of very low affinity that are targeted largely towards disordered regions, persists even in healthy individuals.

**Figure 7:**
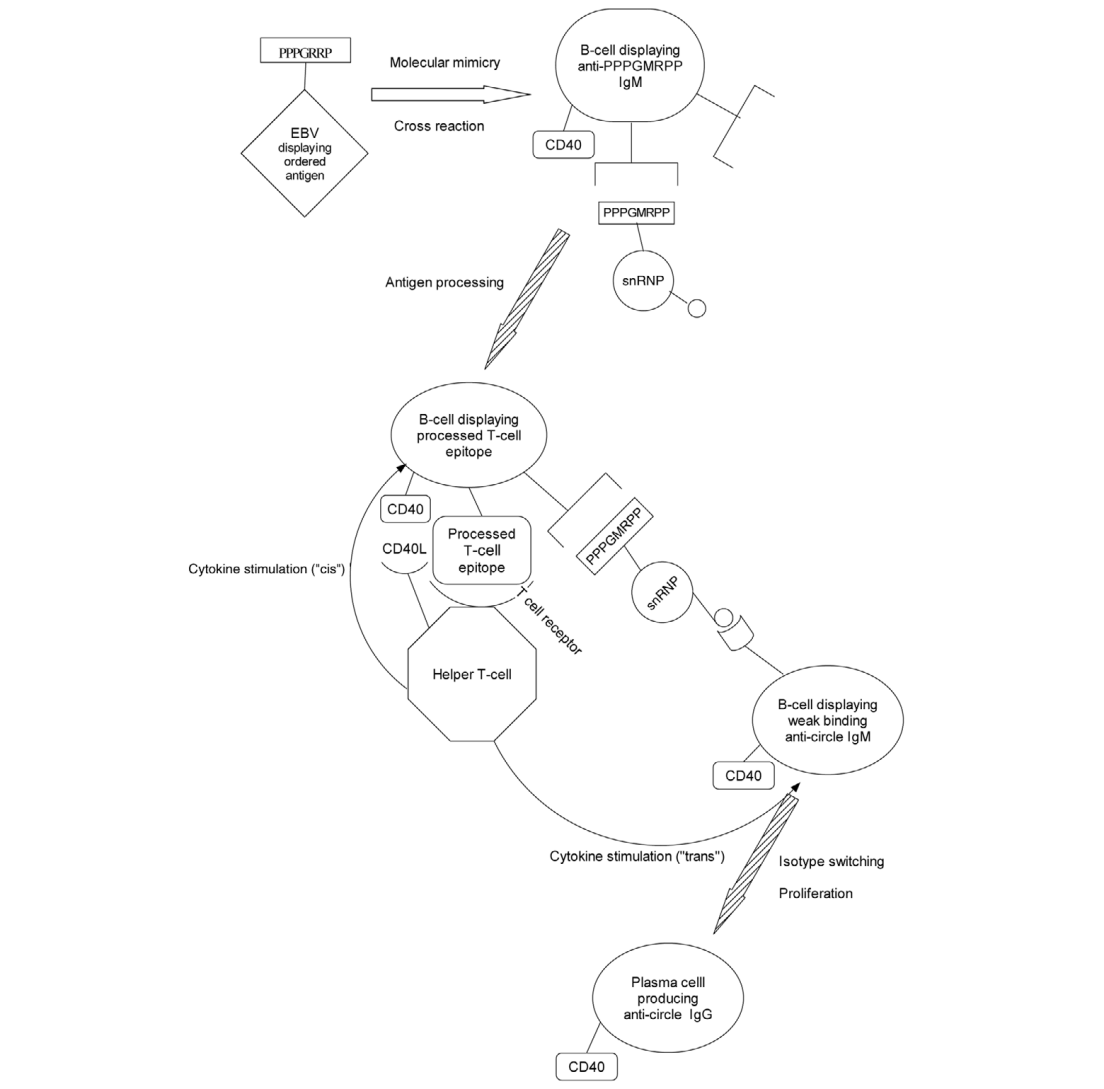
A scaffolding model for antigen spreading. Shown is the target of an autoimmune response; here a snRNP particle that expresses at least two antigenic determinants. The determinant represented by the rectangle, which might be for example the PPPGRRP sequence on EBNA1, is assumed to cross react with the determinant PPPGMRPP on the snRNP via a conventional immune synapse. We denote progenitor B cells participating in these cognate interactions with T cells as primary progenitor B cells. Also shown is a second determinant on the small nuclear ribonucleoprotein particle (snRNP; represented by a circle) that is assumed to be more strongly disordered than the rectangular determinant. Progenitor B cells reacting with this determinant, termed secondary progenitor B cells, are capable of spreading the immune response via an eavesdropping mechanism as discussed in the text. EBV, Epstein-Barr virus.

However, the very success of the concept of autoantigen disorder in explaining the persistence of B cells directed to self-epitopes only intensifies the difficulty of understanding how disordered regions could ever become the targets of autoimmune attack. Having argued that disordered regions are largely invisible to both T and B cells, how can we explain why in a few percent of individuals this invisibility is breached and autoimmune disease ensues? We agree with earlier authors that the key event is likely to be spreading. Although the data presented support the notion that spreading initiates at ordered epitopes and can spread through disordered regions to elicit autoimmune disease, we have said little about how this might occur. What exactly is the role of the ordered epitope in initiating spreading, and how might it contribute to the activation of the pool of self-reactive progenitor B cells potentially targeted to disordered regions? We suggest that a key to this process lies in the large size, high level of expression, and polyvalent nature of most of the nuclear systemic autoantigens and in particular the fact that frequently these autoantigens are part of structural macromolecular complexes. The model is diagrammed in Fig. 7.

In Fig. 7 the 'primary' progenitor B cell displaying the autoreactive surface Ig (sIg) binds to and processes the determinant and displays the resulting epitopes to T cells in the context of MHC II and accessory proteins in a typical immune synapse. We designate this sort of T cell cytokine activation of progenitor B cells 'cis' activation to indicate that the activation is to a progenitor B cell displaying sIg directly in close proximity to a T cell bearing a homologous T cell receptor via a conventional cognate cell to cell immune synapse.

In our view, the 'secondary' B progenitor cell in Fig. 7 becomes activated via a rather different mechanism. B cell progenitors capable of efficient, high-affinity binding to disordered determinants are few. Instead, there are many B cells that bind with low affinity to these determinants, a binding which is insufficient for their deletion or inactivation. The result is the persistence of large numbers of B cells reactive to disordered determinants on proteins. Furthermore, due to the proteolytic lability of strongly disordered peptides, peptides derived from disordered regions cannot be efficiently presented in the context of MHC II, as suggested by the gaps in the T cell epitope profile for disordered regions shown in Figs 4, 5, 6. Thus, it is difficult to present peptides derived from disordered regions in a conventional immune synapse. However, if a second snRNP should bind to a cross reactive B cell progenitor via its own sIg, a 'secondary' B cell progenitor with sIg that binds (weakly) to the circular epitope can be brought into close proximity of the activated T cell, such that cytokines from this T cell, which can act only over short distances, can act in 'trans' to activate the secondary B progenitor cell. This secondary B cell progenitor is thus activated by 'eavesdropping' on the signals being sent from the T cell to the primary B progenitor cell. In this model, the snRNP acts as a molecular scaffold to bring the two B progenitor cells into close proximity of the T cell to allow cytokine eavesdropping.

The model outlined in Fig. 7 bears some resemblance to other published models of spreading [53,54] but it differs in several notable aspects. Fatenejad and Craft [51] propose two models for spreading. In both models, a T cell is able to activate B cells with different specificities, but all the T/B interactions proceed via a conventional immune synapse. This is also the case for the model of Deshmukh *et al*. [52]. The weakness of all these models is that they presuppose secondary B cells binding to and processing secondary antigens in the absence of T cell help. Yet if this process occurred with any frequency, one would expect that such B cells would be activated and subject to immune deletion. The key difference between our model and those proposed is that we do not require such processing by potentially autoimmune B cell precursors because the secondary B cell precursors use 'eavesdropping' to obviate the need for conventional T/B immune synapses. Our model makes several predictions about the nature of the T and B cells that participate in autoimmune disease. Some of these predictions are characteristic of a wide range of models of autoimmunity and are, therefore, not terribly informative in deciding for or against the model. Still, it is important to note that the model is consistent with a great deal of information that is available about autoimmunity, for example, that autoimmune B cell populations are clonal, that the diseases are antigen driven, that the presence of an autoreactive B cell receptor is insufficient in itself to drive that cell into an autoimmune response, that there is no global defect in B cell elimination in autoimmunity and that helper T cells are required for autoimmune disease [55].

More telling are some less obvious predictions. The first of these is that only T cells with a very limited range of specificities are needed to activate secondary B cells carrying a wide range of specificities. The study of T-cell clones specific for several autoantigens of snRNPs strongly supports this prediction [56]. The model also predicts that soluble factors alone are insufficient to drive autoantibody production, and that some of the interactions in systemic autoimmunity are MHC II restricted [57].

Another prediction of the model is that one might find associated with the MHC II protein of secondary B cells peptides that would normally not have access to the MHC II pathway. This breakdown of pathway specificity might occur because there is no T cell synapse to ensure that only class II peptides are presented by the secondary B cells. Such a breakdown in pathway specificity has been experimentally observed [58]. Another prediction is that autoimmunity should be a relatively rare phenomenon on a per cell basis [59]. The model presupposes a syzygy of three immune cells linked via an autoantigen scaffolding. It seems likely that this is a relatively rare event compared to a normal T/B interaction of two cells, but made more likely for highly expressed proteins. The recent observation by Greidinger *et al*. [60] that direct T cell contact with B cells is not needed for T cell help in autoimmunity is also in striking agreement with our model.

Perhaps the most dramatic prediction of the model is that the T cell epitopes associated with autoimmunity should most frequently be derived from ordered regions of autoantigens because such regions can engage in conventional immune synapses. Talken *et al*. [61] have presented evidence supporting this prediction. They identified a series of peptides derived from SmB capable of stimulating T cell clones isolated from patients with SLE. They identified only three T cell epitopes from a total of seven patients. Of these peptides, a single peptide denoted as SmB-E1 comprising residues 16–33 of the SmB sequence was by far the most frequently encountered. This peptide was able to promote the growth of 23 out of 54 total T cell clones. Clones responsive to this peptide were present in five out of the seven patients. Considering that only a single clone was derived from two patients, it is apparent that this epitope is by far the most frequently encountered T cell epitope directed against SmB. A longitudinal analysis showed that response to this epitope remained stable for the two years of the study. Strikingly, this peptide (residues 16–33) is derived from the few residues (17%) in SmB that are predicted to be ordered (Fig. 2d) as predicted by the scaffolding model. We have assembled a considerable amount of additional evidence supporting this prediction (data not shown).

## Conclusion

Nuclear autoantigens exhibit a remarkable degree of disorder. This property may explain the singular skewing of autoantibodies toward these nuclear proteins. We present a framework for considering how protein disorder might lead to autoreactivity. Our scheme unites the notions of tolerance, molecular mimicry, spreading and nucleic acid or protein binding by autoantigens into a coherent whole, but is conservative in that except for introducing the notion of disorder it does not posit any novel attributes of pathogens, the immune system, protein structure or autoantigens that have not been suggested in the past. There are preliminary suggestions that disorder may contribute to the development of autoantigens in the cytoplasm, such as the 60S acidic ribosomal proteins and golgins, and in some types of organ specific disease, such as multiple sclerosis (myelin basic protein), and celiac disease (tissue transglutaminase). Whatever the exact details that emerge from further analysis, we suggest that there is reason to suppose that protein order/disorder has a part to play in explaining the *Wunderkammer *of autoantigens.

## Abbreviations

EBV = Epstein-Barr virus; hNNuSP = human non-nuclear protein database; hNuSP = human nuclear protein database; hNuSysAAG = human nuclear systemic autoantigen database; hSP = human protein database; LDR = long disordered region; MHC = major histocompatibility complex; sIg = surface Ig; SLE = systemic lupus erythematosus; snRNP = small nuclear ribonucleoprotein particle.

## Competing interests

The authors declare that they have no competing interests.

## Authors' contributions

DL Carl and DL Cohen are responsible for most of the analysis, interpretation, and writing of the manuscript. BRST contributed invaluable informatics and statistical input as well as suggesting key aspects of the scaffolding model.

## Supplementary Material

Additional file 1 An Excel file containing a table listing the human nuclear systemic autoantigens in our study, along with their Swiss-Prot accession number, associated disease and length of all disordered regions of more than 20 residues. Click here for file
